# Corticotropin Releasing Factor-Induced CREB Activation in Striatal Neurons Occurs via a Novel Gβγ Signaling Pathway

**DOI:** 10.1371/journal.pone.0018114

**Published:** 2011-03-23

**Authors:** Christopher M. Stern, Jessie I. Luoma, John Meitzen, Paul G. Mermelstein

**Affiliations:** 1 Graduate Program in Neuroscience, University of Minnesota, Minneapolis, Minnesota, United States of America; 2 Department of Neuroscience, University of Minnesota, Minneapolis, Minnesota, United States of America; University of California, Berkeley, United States of America

## Abstract

The peptide corticotropin-releasing factor (CRF) was initially identified as a critical component of the stress response. CRF exerts its cellular effects by binding to one of two cognate G-protein coupled receptors (GPCRs), CRF receptor 1 (CRFR1) or 2 (CRFR2). While these GPCRs were originally characterized as being coupled to Gα_s_, leading to downstream activation of adenylyl cyclase (AC) and subsequent increases in cAMP, it has since become clear that CRFRs couple to and activate numerous other downstream signaling cascades. In addition, CRF signaling influences the activity of many diverse brain regions, affecting a variety of behaviors. One of these regions is the striatum, including the nucleus accumbens (NAc). CRF exerts profound effects on striatal-dependent behaviors such as drug addiction, pair-bonding, and natural reward. Recent data indicate that at least some of these behaviors regulated by CRF are mediated through CRF activation of the transcription factor CREB. Thus, we aimed to elucidate the signaling pathway by which CRF activates CREB in striatal neurons. Here we describe a novel neuronal signaling pathway whereby CRF leads to a rapid Gβγ- and MEK-dependent increase in CREB phosphorylation. These data are the first descriptions of CRF leading to activation of a Gβγ-dependent signaling pathway in neurons, as well as the first description of Gβγ activation leading to downstream CREB phosphorylation in any cellular system. Additionally, these data provide additional insight into the mechanisms by which CRF can regulate neuronal function.

## Introduction

Stress is any actual or perceived disturbance of an organism's environment. An acute stress response, which includes the release of corticotropin-releasing factor (CRF), is often necessary for the preservation of organismal integrity during brief anxiety situations. CRF binding to its cognate G-protein coupled receptors (GPCRs), CRF receptor 1 (CRFR1) and 2 (CRFR2), mediates the influence of CRF on brain cells. However, in addition to their beneficial role in an acute stress response, both stress and CRF have been implicated in pathological disease states, including drug addiction.

Several lines of evidence suggest that stress, and CRF in particular, influence addiction and addictive behaviors. Drugs of abuse have been shown to activate the hypothalamic-pituitary-adrenal (HPA) axis [1], initiating the stress response. Human individuals with a history of chronic stress are more likely to become addicts, and previously abstinent addicts are more likely to relapse following exposure to an acute stressor [1]–[3]. A stress event both increases drug-seeking behavior as well as facilitates conditioned-place preference to drugs of abuse in animal models of addiction [4]–[10]. Furthermore, stress and CRF potentiate the rewarding effects of drugs of abuse [11].

Recent findings suggest that CRF-induced CREB phosphorylation within the nucleus accumbens (NAc) underlies at least some of these effects of stress on addictive behaviors [11]. As CREB signaling in NAc is critical for the rewarding actions of drugs of abuse [12]–[16], CRF activation of CREB represents a putative molecular mechanism by which stress could manipulate the neural circuitry underlying drug addiction.

Not surprisingly, the effects of CRF on NAc functioning are not limited to actions related to drug abuse, but also affect the rewarding actions of more physiological stimuli. For example, CRF acting in NAc plays an essential role in prairie-vole pair bonding [17], as well as enhances the incentive salience of a sucrose reward [18]. And while research thus far has focused on the NAc, CRF neurotransmission is also an essential component to connectivity in the dorsal striatum [19]–[22].

Although the influence of CRF on striatal functioning has been established, the molecular mechanisms by which this occurs remain unclear. Given the known relevance for CRF activation of CREB, we characterized the intracellular signaling pathway by which this occurs. While CRFRs are classically thought of as Gα_s_-coupled GPCRs that exert their effects via activation of adenylyl cyclase (AC) and subsequent increases in the second messenger cAMP [23], [24], these receptors have since been shown to couple to multiple G-protein signaling cascades in neurons [25]. Through use of pharmacological and genetic approaches, we report a novel signaling pathway whereby CRF leads to a rapid Gβγ-dependent increase in CREB phosphorylation: an effect mediated by MAPK signaling. In addition to illuminating the pathways by which CRF affects striatal neurons, this is the first example of CRF leading to downstream Gβγ-dependent signaling, as well as the first example of Gβγ activation leading to CREB phosphorylation.

## Results

### CRF Induces Rapid CREB Phosphorylation via Activation of CRFR1

**Figure 1:**
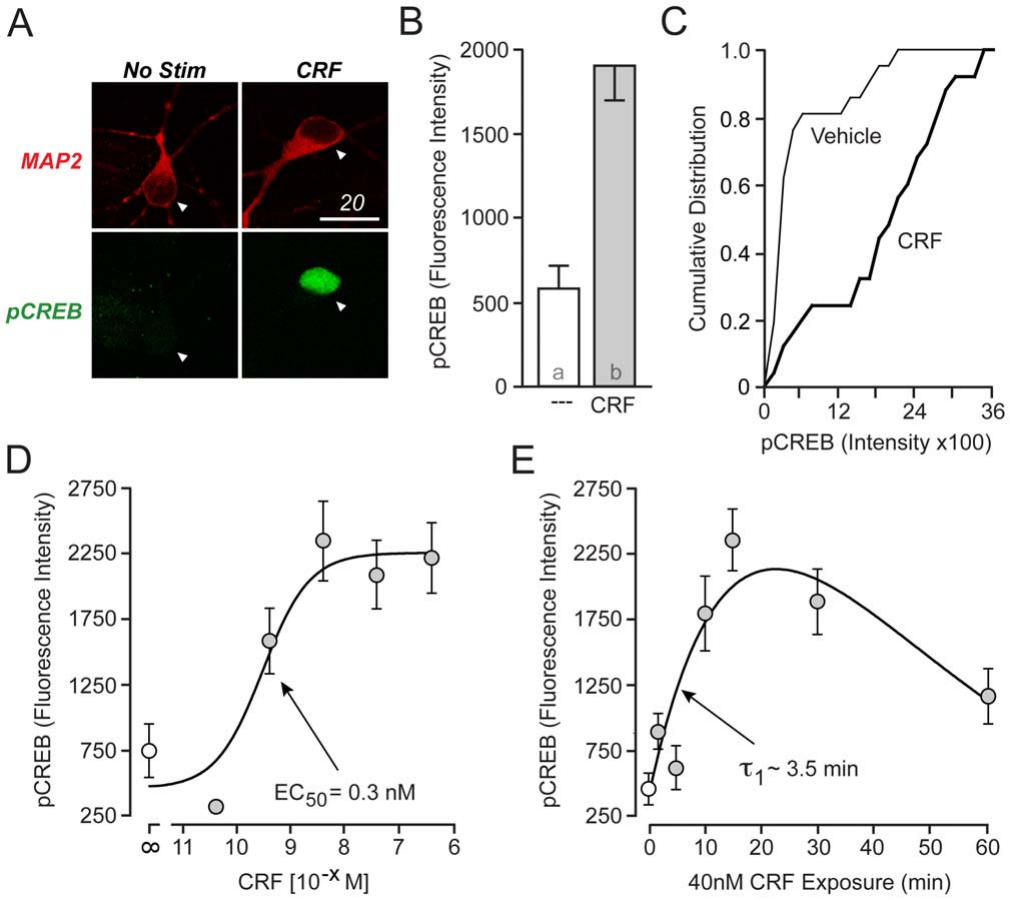
CRF rapidly stimulates CREB phosphorylation. (A) Immunolabeled confocal images of cultured striatal neurons from 1- to 2-day old rat pups with MAP2 (red) and pCREB (green). Neurons stimulated with CRF (40 nM) for 15 min exhibited increased CREB phosphorylation (Scale Bar  =  20 µm). (B) Quantification of immunostaining, revealing CRF-mediated CREB phosphorylation (p<0.0001). (C) CRF induced a rightward shift in the plot of pCREB fluorescence intensity of approximately 80% of striatal neurons (D) CRF increased CREB phosphorylation in a concentration-dependent manner, with EC_50_  =  0.3 nM. Concentrations ≥ 4 nM induced a signal that differed with statistical significance from vehicle-stimulated (NS) neurons. (E) Time course of 40 nM CRF-induced CREB phosphorylation, with τ  =  3.5 min. Statistically different groups are denoted by different alphabetical characters in corresponding bar graphs in this and subsequent figures. P-values <0.05 were considered *a priori* as significant.

Stress-induced activation of CREB in the striatum requires CRFR1 activation [11]. Thus, we attempted to determine whether CRF can lead to CREB phosphorylation (pCREB) in cultured striatal neurons from 1- to 2-day old rat pups, and if so, through which molecular pathway(s). Indeed, a 15-min application of CRF (40 nM) significantly increased CREB phosphorylation relative to vehicle-stimulated control neurons (Figure 1), but had no effect on total CREB staining (Figure S1). When measuring pCREB fluorescence, CRF produced an observable shift in the population response of striatal neurons (Figure 1C). Specifically, plotting the pCREB data as a cumulative histogram revealed that, CRF produced a large shift in pCREB fluorescence intensity in approximately 80% of the striatal neurons (Figure 1C). Furthermore, CRF increased CREB phosphorylation in a concentration-dependent manner with an EC_50_  =  0.3 nM (Figure 1D), consistent with a CRFR-mediated event (binding affinity of CRF for CRFR1 ∼ 5–10 nM [26]). CRF-induced increases in pCREB occurred in a rapid and time-dependent manner (τ∼3.5 minutes), with extended exposure (1 hr) producing apparent desensitization (Figure 1E).

The striatum is composed of two major sub-regions, the dorsal striatum (i.e. caudate/putamen) and nucleus accumbens (NAc). To determine whether CRF-induced CREB phosphorylation is restricted to neurons from one of these two anatomical regions, we created dorsal striatum “enriched” and NAc “enriched” cultures. Both cultures displayed similarly robust increases in CREB phosphorylation upon CRF application (data not shown), suggesting that there are no overt differences in CRF-responsiveness between these two brain regions. Thus, we performed our remaining experiments in whole striatal cultures.

**Figure 2:**
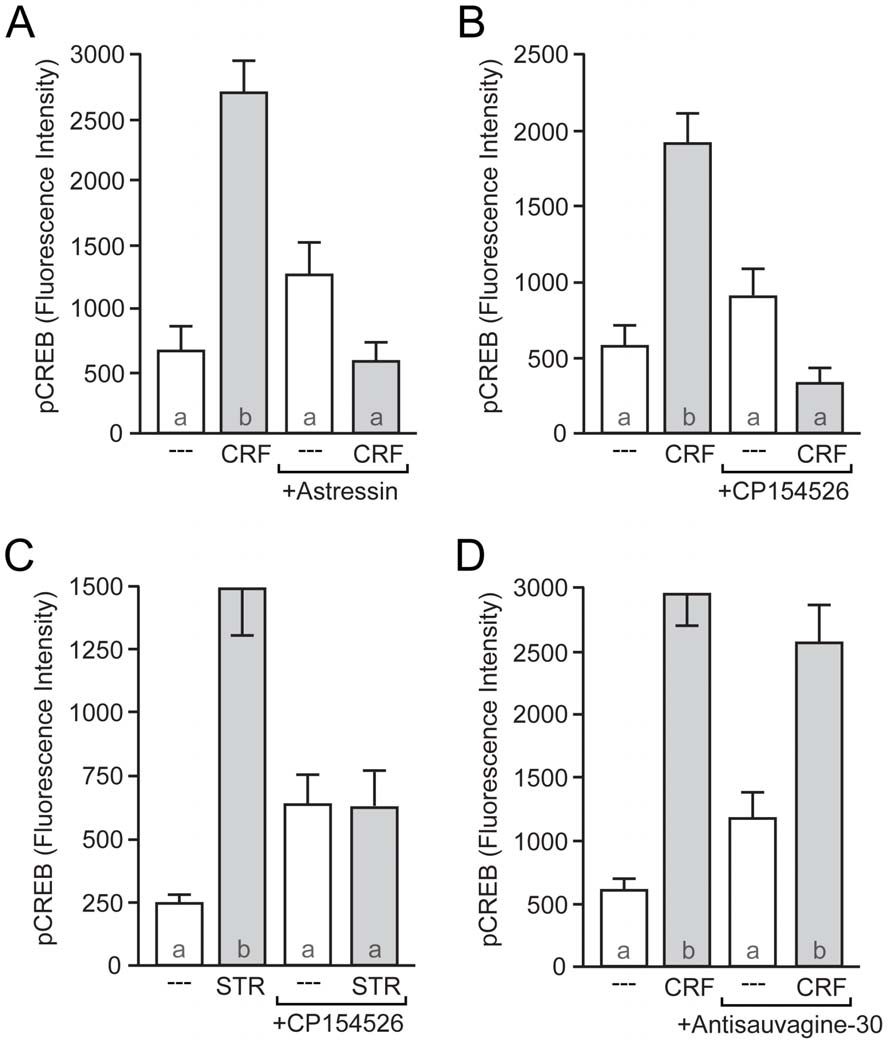
CRFR1 mediates CRF-induced CREB phosphorylation. (A) CRF-mediated CREB phosphorylation was blocked by the non-specific CRFR antagonist astressin (50 nM; *F* = 20.19), (B) and the CRFR1-specfic antagonist CP154526 (100 nM; *F* = 17.40). (C) CRF-induced CREB phosphorylation was mimicked by the CRFR1 agonist stressin-1 (STR; 70 nM). STR-induced CREB phosphorylation was also blocked by CP154526 (*F* = 15.99). (D) The CRFR2-specific antagonist antisauvagine-30 (100 nM) had no effect on CRF-induced CREB phosphorylation (*F* = 17.10).

The striatum expresses both CRFRs, although CRFR1 to a greater extent than CRFR2 [27]–[30]. We confirmed expression of both receptors in our cultured neuronal system using real-time PCR (data not shown). To determine whether CRF-induced CREB phosphorylation occurs via activation of these classic CRFRs, we pre-incubated our striatal neurons with the non-specific CRFR peptide antagonist astressin (50 nM). Indicative of a CRFR-mediated event, this treatment completely abolished CRF-induced CREB phosphorylation (Figure 2A). Next, we sought to determine which receptor mediates CRF-induced CREB phosphorylation. Pre-incubation with the specific CRFR1 antagonist CP154526 (100 nM) eliminated CRF-induced CREB phosphorylation (Figure 2B). In addition, a 15 min application of the CRFR1-specific agonist stressin-1 (STR; 70 nM) mimicked the effect of CRF, and this effect was also blocked with CP154526 (Figure 2C). To determine any putative role of CRFR2 in CRF-induced CREB phosphorylation, we utilized two CRFR2-preferring peptide antagonists. Both antisauvagine-30 (100 nM; Figure 2D) and K41498 (10 nM) (not shown) failed to block CRF-induced CREB phosphorylation. Together, these data strongly demonstrate that CRF induced CREB phosphorylation in striatal neurons is mediated exclusively through CRFR1, with CRFR2 playing no discernable role.

### CRF Does Not Induce CREB Phosphorylation via the AC/cAMP/PKA Pathway

**Figure 3:**
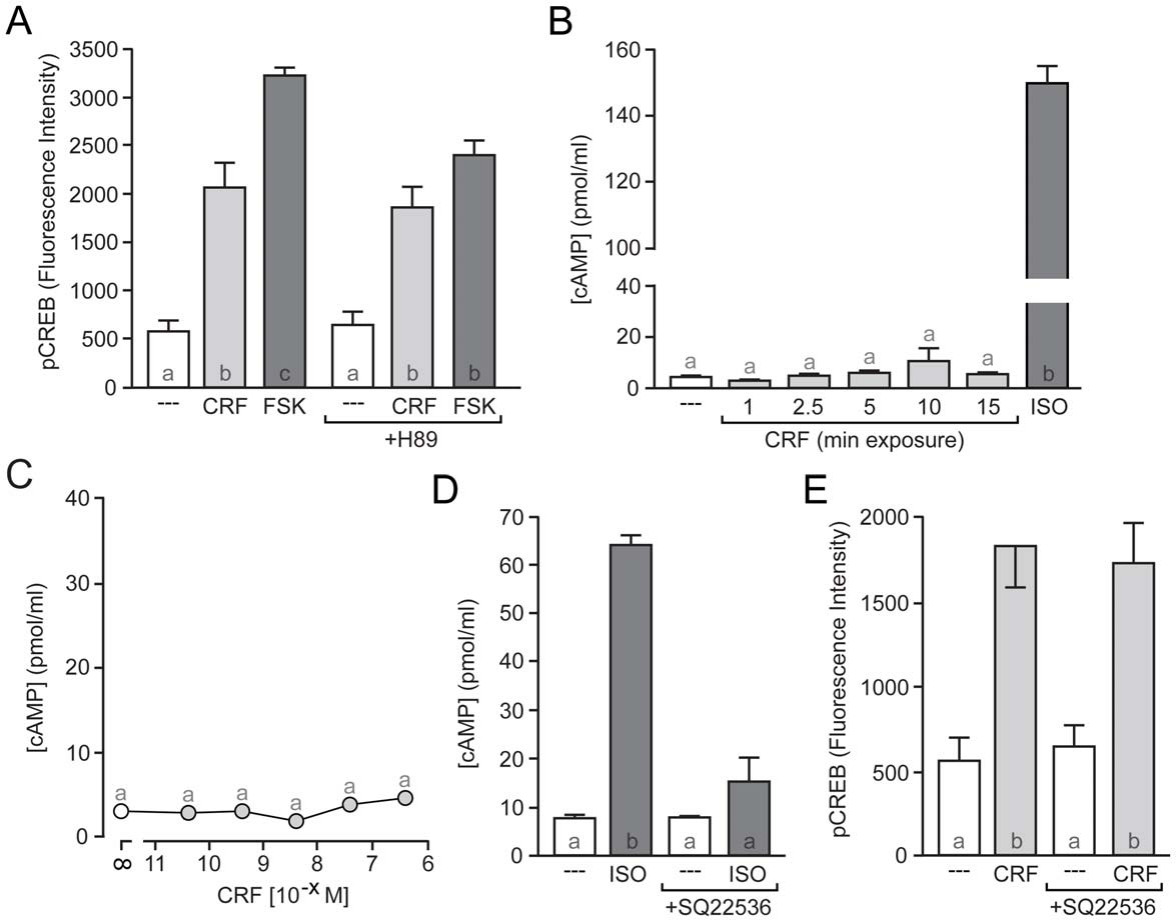
CRF-induced CREB phosphorylation occurs independently of the AC/cAMP/PKA pathway. (A) A PKA-specific concentration of the protein kinase inhibitor H89 (2 µM) failed to block CRF-induced CREB phosphorylation, but attenuated forskolin (FSK)-induced CREB phosphorylation (*F* = 35.78). (B) In the presence of the phosphodiesterase inhibitor IBMX (75 µM), CRF (40 nM) failed to increase cAMP during the time-course in which it increases pCREB. The β-adrenergic receptor agonist isoproterenol (ISO; 10 µM) was used as a positive control (*F* = 3226). (C) Several concentrations of CRF up to and including 400 nM failed to increase cAMP accumulation (in the presence of IBMX). (D) Inhibiting AC activity with SQ22536 (90 µM) completely blocked ISO-induced cAMP accumulation (*F* = 101.4). (E) Inhibition of AC with SQ22536 had no effect on CRF-induced CREB phosphorylation (*F* = 17.10).

CRFR1 is primarily thought of as a GPCR that signals through Gα_s_ to produce subsequent increases in cAMP. Hence, we decided first to test the hypothesis that CRF activation of CRFR1 leads to an AC/cAMP/PKA-dependent increase in CREB phosphorylation. To assay for the involvement of this pathway, we began with inhibition of PKA. A PKA-specific concentration [31], [32] of the protein kinase inhibitor H-89 (2 µM) failed to block CRF-induced CREB phosphorylation (Figure 3A). Importantly, this concentration of H89 attenuated CREB phosphorylation stimulated by the AC agonist forskolin (FSK; 25 µM), which signals to CREB through multiple cAMP-responsive proteins including PKA (Figure 3A). Given this unexpected result, we attempted to block CRF-induced CREB phosphorylation with three additional PKA antagonists: the cell permeable peptide PKA inhibitor PKI, 14–22 amide (1 µM), KT5720 (1 µM), and cAMPS-Rp, triethylammonium salt (10 µM). All three compounds also failed to eliminate CRF-induced CREB phosphorylation (data not shown). Together, these data demonstrate that PKA is not involved in CRF-induced CREB phosphorylation in striatal neurons.

We next determined whether CRF was in fact eliciting increases in cAMP under conditions in which CRF was producing CREB phosphorylation. Hence, we quantified cAMP concentrations following the CRF stimulation paradigm used to elicit increases in CREB phosphorylation. To prevent potential loss of the cAMP signal during the agonist stimulations, the phosphodiesterase inhibitor IBMX (75 µM) was included both prior to and during stimulation. Exposure of striatal cultures to CRF (40 nM) for durations that elicited CREB phosphorylation failed to induce any measurable increase in cAMP concentration (Figure 3B). In fact, stimulation of striatal cultures for 15 min with various concentrations of CRF up to and including 400 nM (Figure 3C) failed to induce a significant accumulation of cAMP. As a positive control for our assay [33], a 15 min application of the β-adrenergic receptor agonist isoproterenol (ISO; 10 µM) resulted in a significant increase in cAMP (Figure 3B).

To directly test whether cAMP signaling played a role in CRF-induced CREB phosphorylation, we treated striatal cultures with the AC antagonist SQ22536 (90 µM). While SQ22536 completely blocked ISO-induced cAMP formation (Figure 3D), it failed to affect CRF-induced CREB phosphorylation (Figure 3E). Together, these data demonstrate that rapid CRF-induced CREB phosphorylation in striatal neurons operates independently of AC, cAMP, and PKA signaling.

### CRF Induces CREB Phosphorylation via Calcium-Independent MAPK Signaling

**Figure 4:**
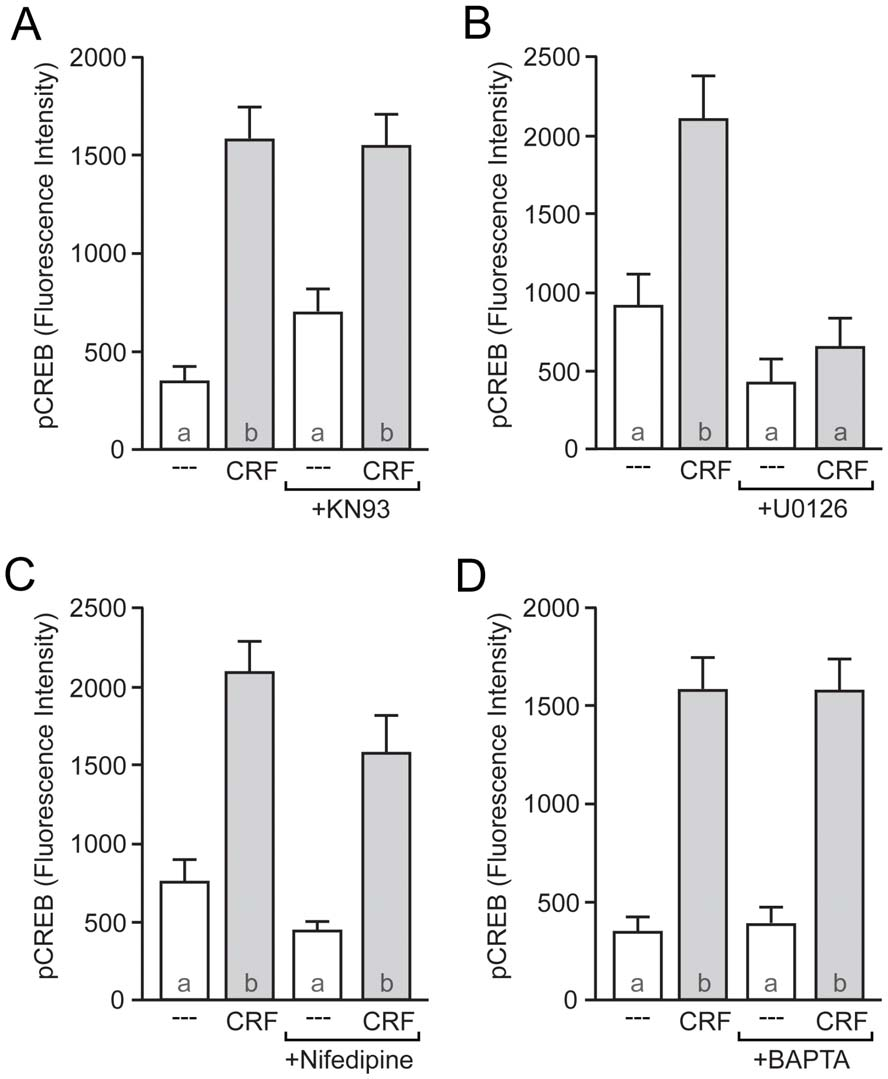
CRF-induced CREB phosphorylation occurs via a MEK/MAPK-dependent mechanism. (A) The CaMK inhibitor KN-93 (2 µM) did not affect CRF-induced CREB phosphorylation (*F* = 17.65). (B) CRF-induced CREB phosphorylation was blocked by the MEK antagonist U0126 (10 µM; *F* = 11.97). (C) The L-type Ca^2+^ channel blocker nifedipine (5 µM) had no effect on CRF-induced CREB phosphorylation (*F* = 18.50). (D) Chelating intracellular calcium with BAPTA-AM (30 min incubation with 5 µM) failed to block CRF-induced CREB phosphorylation (*F* = 12.72).

Independent of PKA, several downstream kinase pathways have been shown to regulate CREB phosphorylation [34], [35]. One such kinase is CaMKIV. However, blocking CaMK activation with KN-93 (2 µM) had no effect on CRF-induced CREB phosphorylation (Figure 4A), although this concentration of KN-93 blocks depolarization-induced CREB phosphorylation [36].

MAPK signaling is another pathway that has been shown capable of CREB activation in a variety of neuronal preparations. In contrast to previous experiments, the MEK (MAPK kinase) inhibitor U0126 (10 µM) blocked CRF-induced CREB phosphorylation (Figure 4B). A second MEK antagonist, PD98059 (25 µM), yielded a similar result (data not shown). MAPK-dependent CREB phosphorylation is often calcium dependent, following calcium entry through L-type calcium channels, or release of calcium from intracellular stores. However, CRF-mediated CREB phosphorylation was not affected by either the L-type calcium channel blocker nifedipine (5 µM; Figure 4C) or by chelating intracellular calcium through pre-incubation with BAPTA-AM (Figure 4D). Removing extracellular calcium during CRF stimulation also had no effect on CRF-mediated CREB phosphorylation (data not shown). Thus, CRF activation of MAPK signaling appears to be calcium independent.

### CRF Induces CREB Phosphorylation via a Gβγ-Dependent Mechanism

We next decided to test the involvement of several upstream signalers that could lead to downstream MAPK activation, including Akt, receptor tyrosine kinases (RTKs), Ras, Raf, ROCK, and PI3K. We utilized pharmacological blockers for each of these molecules to test for any contribution in CRF-induced CREB phosphorylation. Remarkably, inhibitors of Akt (10-DEBC; 5 µM), RTKs (K252A; 100 nM), Ras (XRP44X; 2 µM), Raf (GW5074; 1 µM) ROCK (H-1152; 500 nM), and PI3K (wortmannin; 500 nM) all failed to affect CRF-induced CREB phosphorylation (data not shown). Unable to identify an intermediary signaling molecule upstream of MEK but downstream of CRFR1, we decided to test for the involvement of different G-protein subunits in CRF-induced CREB phosphorylation.

**Figure 5:**
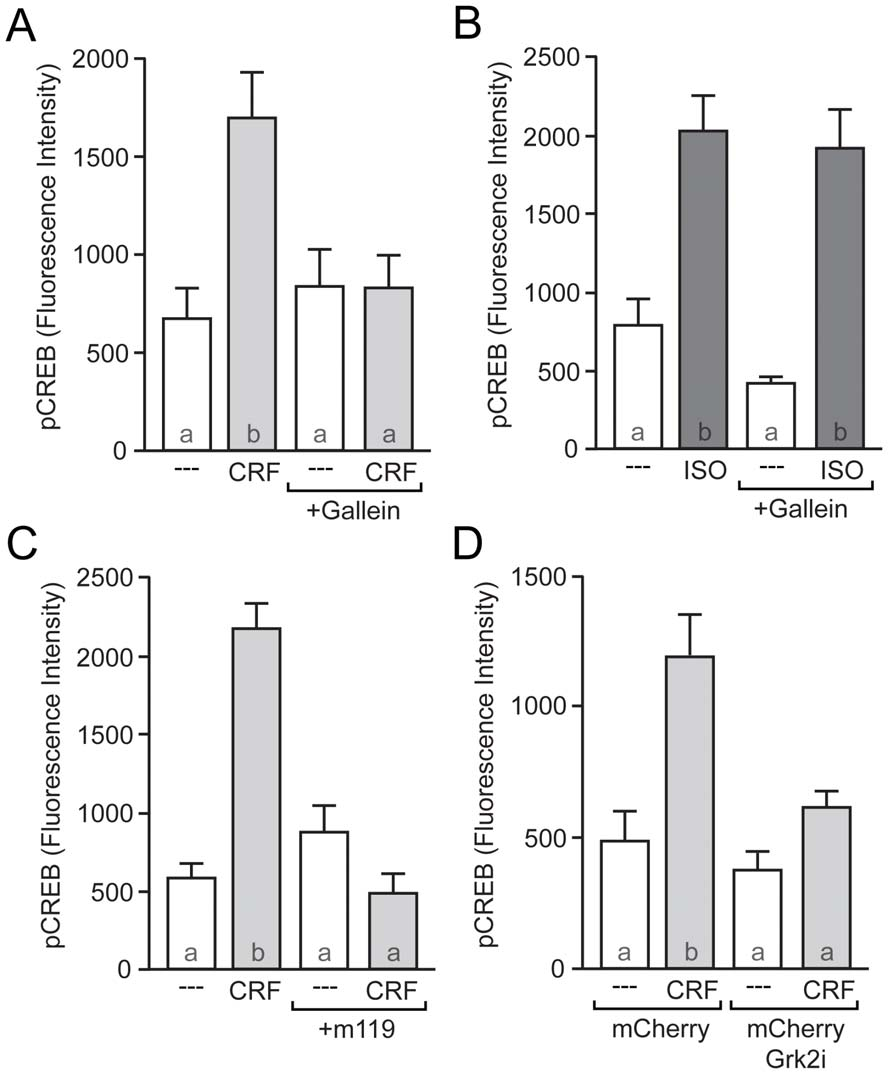
CRF-induced CREB phosphorylation occurs via a Gβγ-dependent mechanism. (A) The Gβγ-specific blocker gallein (75 µM) eliminated CRF-induced CREB phosphorylation (*F* = 6.13). (B) In contrast, gallein had no effect on CREB phosphorylation induced by the β-adrenergic receptor agonist, isoproterenol (*F* = 16.83). (C) An additional Gβγ blocker (M119; 5 µM) also eliminated CRF-induced CREB phosphorylation (*F* = 27.96). (D) Neurons transfected mCherry and the Gβγ inhibitory peptide Grk2i failed to exhibit CRF-induced CREB phosphorylation (*F* = 11.26).

To test for a Gβγ-dependent mechanism in CRF-induced CREB phosphorylation, we utilized the Gβγ-specific inhibitor gallein (75 µM) [37]. In support of a role for Gβγ, CRF failed to induce CREB phosphorylation in the presence of this drug (Figure 5A). To ensure that the effect of gallein was specific to CRF and not dampening all G-protein signaling, we stimulated our neurons with ISO (10 µM) in the presence of gallein. Since ISO leads to CREB phosphorylation via a Gα_s_ pathway in striatal neurons [33], gallein should not affect ISO-dependent CREB phosphorylation. Indeed, this was in fact the case (Figure 5B). To verify our initial results, we blocked CRF-induced CREB phosphorylation with a second Gβγ-specific inhibitor, M119 (5 µM; Figure 5C). Again M119 had no effect on ISO-induced CREB phosphorylation (data not shown; also see [33]).

To complement these pharmacological data, we utilized a genetic approach to further characterize the involvement of Gβγ in CRF-induced CREB phosphorylation. Neurons were transfected with either a construct expressing mCherry or co-transfected with the mCherry construct plus a construct expressing the peptide motif of G-protein regulated kinase that scavenges Gβγ (Grk2i), which has previously been shown to specifically block downstream Gβγ signaling [38]. Neurons that were transfected with mCherry alone demonstrated robust CRF-induced CREB phosphorylation. However, neurons that were transfected with Grk2i failed to show CRF-induced CREB phosphorylation (Figure 5D). Together, these pharmacological and genetic data support the hypothesis that CRF-induced CREB phosphorylation is mediated via a Gβγ-dependent signaling mechanism.

### CRFR1 Couples to Gα_s_ in Striatal Neurons

**Figure 6:**
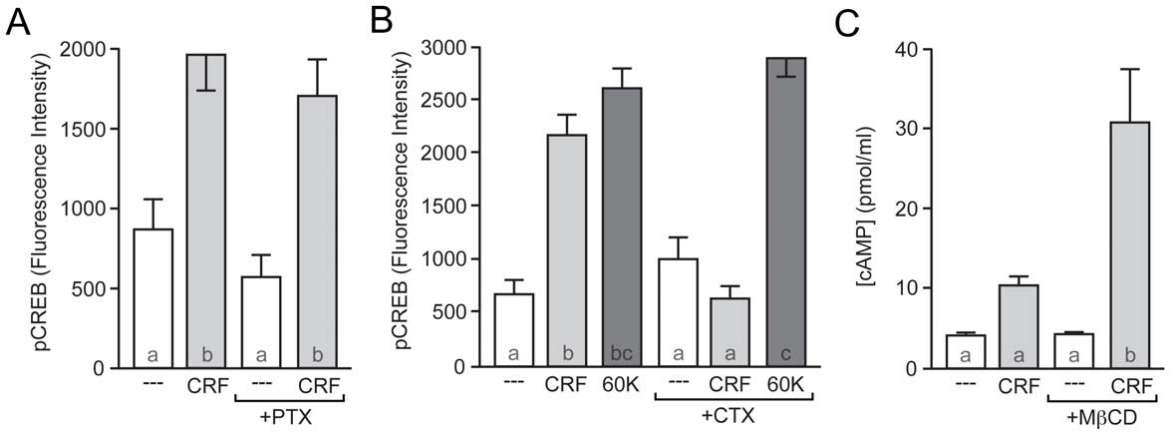
CRFR1 couples to Gα_s_ in striatal neurons. (A) Pre-treatment with pertussis toxin (PTX; 500 ng/mL) did not affect CRF-induced pCREB (*F* = 11.10). (B) CTX blocked CRF-induced CREB phosphorylation, but had no effect on MAPK-dependent CREB phosphorylation induced by depolarization (60 K; *F* = 35.15). (C) CRF (4 µM) increased cAMP concentrations in the presence of the cholesterol chelator methyl-β-cyclodextrin (MβCD; 10 mM).

To identify the Gα subunit to which CRFR1 and Gβγ couple in this paradigm, we utilized toxins that specifically target functional classes of Gα. Since functional Gβγ subunits in neurons are classically thought to associate with Gα_i/o_, we first utilized pertussis toxin (PTX) to specifically inhibit these Gα subunits. PTX prevents the exchange of GDP (inactive state) for GTP (active state), thus hindering the G-protein trimer (Gαβγ) from dissociating from the GPCR, and initiating downstream signaling. Overnight (18 hr) pretreatment of striatal neurons with PTX (0.5 µng/µL) did not alter CRF-mediated CREB phosphorylation (Figure 6A).

Next, we targeted the Gα_s_ family, which includes Gα_s_ and Gα_olf_ in the striatum [39] (hereafter termed Gα_s_), with cholera toxin (CTX). CTX blocks inactivation of Gα_s_ by preventing the hydrolysis of GTP (active state) to GDP (inactive state). A short term (2 hr) pre-treatment with CTX induced a transient increase in pCREB on its own (NS: 397.7±93.5; CTX: 2809.7±104.5), confirming that Gα_s_ can signal to CREB in this paradigm. However, to test the effect of disrupting Gα_s_ signaling on CRF-induced CREB phosphorylation, we used long-term CTX treatment, which allows the pCREB signal to reset, as well as prevents Gα_s_ from re-associating with either the Gβγ subunits or the GPCR, precluding agonist-induced reactivation of this signaling pathway. Indeed, overnight pretreatment with CTX resulted in a complete block of CRF-induced CREB phosphorylation (Figure 6B). Importantly, MAPK-mediated CREB phosphorylation following a 60 mM K^+^ depolarization [36] was unaffected by this treatment (Figure 6B), indicating signaling downstream of CRFR1 was still intact.

Since AC is the primary downstream effector of Gα_s_, and since CRFR1 couples to Gα_s_ in striatal neurons (Figure 6), we wondered why we failed to observe CRF-induced cAMP accumulation (Figure 3). We hypothesized that CRFR1 is in fact organized into discrete membrane lipid microdomains [40], which (1) facilitate Gβγ activation of downstream MAPK signaling and (2) prevents Gα_s_ from accessing/stimulating AC. Therefore, we hypothesized that disrupting lipid microdomains would allow Gα_s_ to activate AC following CRF stimulation. To test this hypothesis we pre-incubated our striatal neurons with the cholesterol chelator methyl-β-cyclodextrin (MβCD; 10 mM), commonly used to disrupt lipid microdomains [41], [42]. We found that unlike control conditions, 4 µM CRF induced a significant increase in cAMP formation in the presence of MβCD (Figure 6C), although notably, not to the levels seen with isoproterenol (see discussion). These data are consistent with the hypothesis that CRF is liberating Gα_s_ subunits in order to mediate Gβγ-mediated CREB phosphorylation.

## Discussion

### CRF Increases CREB Phosphorylation via a Novel Signaling Pathway

CRF signaling in neurons was initially characterized as being mediated via the Gα_s_-coupled CRFR1, and subsequent downstream activation of AC, cAMP and PKA. However, given the recent evidence for the promiscuity of CRF coupling to intracellular signaling pathways, and the profound influence of CRF on NAc processes via CREB, our experiments were designed to determine the specific signaling pathway by which CRF-induced CREB phosphorylation in striatal neurons. Using a combination of pharmacological and genetic approaches, we report a novel neuronal signaling pathway whereby CRF binds to a Gα_s_-coupled CRFR1, leading to a Gβγ- and MAPK-dependent increase in CREB phosphorylation. Notably, these data are consistent with previous findings showing that stress-facilitation of drug reward depends on CRFR1 and CREB activation in NAc [11], as well as the finding that CRFR1 is the predominant CRF receptor expressed within this brain region [27]–[30].

These data demonstrating CRF signaling through a non-canonical pathway are consistent with numerous reports of CRFRs coupling to diverse intracellular signaling pathways across distinct neuronal populations. CRF has been shown to activate CREB in cerebellar and hippocampal neurons [43], as well as MAPKs in several brain regions [44]. Separate from CREB/MAPK signaling, CRF also affects the activity of various other kinases, second messengers, ion channels and neurotransmitter receptors [45]–[51]. This diversity in cellular effects is not surprising when one considers the ability of CRF to influence a variety of natural and pathological behaviors [17], [52]–[54]. However, this report is unique among papers describing CRF signaling in neurons in that it describes a novel upstream signaling activator at the level of the G-protein subunit (Gβγ).

Although this is the first report demonstrating that Gβγ leads to CREB activation, and that CRF and CRFR1 signal via Gβγ in neurons, there have been reports of CRF acting through Gβγ-dependent mechanisms in cell lines expressing recombinant protein. These include findings that CRF effects on T-type calcium channels [55] and intracellular calcium signaling [56] depend on Gβγ function, as well as a study that found that Gβγ is important for CRFR desensitization [57]. There is also a precedent for activation of Gβγ leading to downstream MAPK activation in non-neuronal cell lines [58]–[60]. One group has shown indirect evidence for Gβγ mediated CREB activation in cerebellar granule neurons [61] by demonstrating (1) that Gβγ subunits mediate GABA_B_ receptor-induced ERK activation, and (2) that GABA_B_-mediated CREB phosphorylation is sensitive to MEK/ERK inhibition. Importantly, the authors did not demonstrate in their pCREB assay that GABA_B_-induced CREB phosphorylation is sensitive to Gβγ inhibition. Hence, our approach is unique in that it is the first report using a direct assay of CREB phosphorylation to demonstrate that agonist-induced increases in pCREB are sensitive to inhibition of Gβγ signaling. Given the diversity of Gβγ signaling [58], [60] and the plethora of CRF effects in numerous brain regions and peripheral tissues [25], future studies can test for a role of Gβγ signaling in CRF function throughout the brain and other tissue.

**Figure 7:**
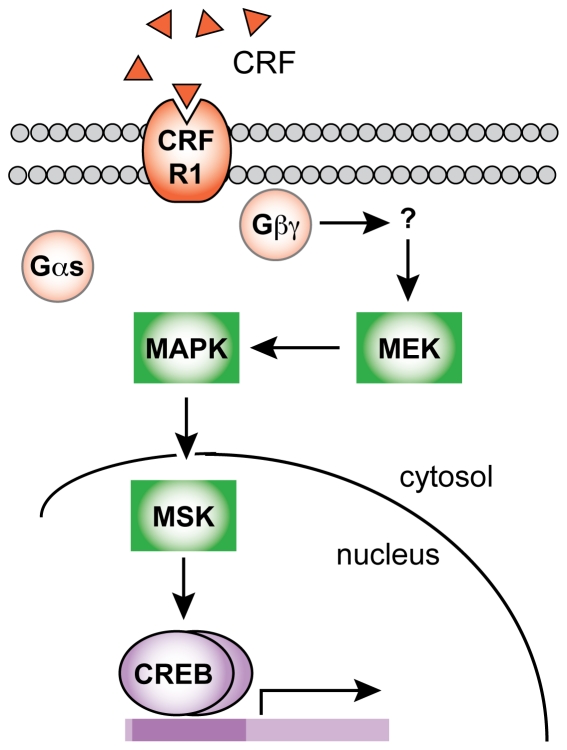
Proposed mechanism of CRF-induced CREB phosphorylation in striatal neurons. CRF binds to Gα_s_-coupled CRFR1, leading to a Gβγ-dependent activation of MEK and MAPK, resulting in downstream CREB phosphorylation.

The mechanism by which Gβγ leads to MAPK signaling in striatal neurons remains elusive [59], [60]. One hypothesis is that an intermediate signaling moiety couples Gβγ to MEK (Figure 7). We assayed for the involvement of signaling molecules that have previously been shown to lead to downstream MAPK/CREB activation, are known to be activated by Gβγ, or both. Pharmacological inhibitors/blockers of L-type calcium channels, CaMK, AKT/PKB, RTKs, ROCK, Ras, Raf, and PI3K all failed to block CRF-induced CREB phosphorylation. Furthermore, we ruled out any contribution of calcium to CRF induced CREB phosphorylation in these neurons.

Alternatively, Gβγ could physically interact with and activate MEK to initiate MAPK signaling. Gβγ has been shown to interact with and activate a variety of downstream signaling molecules, including AC and ion channels [60]. However, MEK/MAPK are not among those known be physically regulated by Gβγ activation. Interestingly, Gβγ has been shown to traffic to cytoplasmic sites distinct from the plasma membrane, including the Golgi complex and the endoplasmic reticulum [62], [63]. It is unknown whether receptor-mediated activation of Gβγ subunits can transduce cytoplasmic signaling via translocation into the cytoplasm. However, the hypothesis that Gβγ activated at the membrane can translocate to the cytoplasm to lead to MEK activation cannot be ruled out. Although beyond the scope of this study, future investigations in both neuronal and non-neuronal cells will be needed to determine the exact mechanism by which Gβγ leads to downstream MEK/MAPK activation.

Independent of the mechanism by which Gβγ activates downstream signaling in this paradigm, we hypothesize that the Gβγ subunits released from CRFR1 in response to ligand binding are unique in their ability to elicit downstream CREB phosphorylation, insofar as dissociation of Gβγ from any GPCR is insufficient for this effect. For instance, ISO-mediated activation of β-adrenergic receptors does not result in Gβγ-mediated CREB phosphorylation (Figure 5B). One hypothesis is that CRFR1-coupled Gβγ subunits have a unique molecular property (i.e. post-translational modification) that facilitates activation of downstream signaling pathways. However, our data with the cholesterol chelator MβCD (Figure 6C) suggest an alternative hypothesis whereby the sub-cellular localization of CRFR1 in discrete lipid microdomains allows its dissociated Gβγ subunits privileged access to the downstream signaling machinery necessary for CREB phosphorylation, while preventing CRFR1-associated Gα_s_ from accessing AC. Indeed, an emerging theme in neuronal intracellular signaling is the hypothesis that both membrane-associated and intracellular signaling proteins are organized into discrete structural and functional microdomains that facilitate efficacious intracellular signaling [40]. There is precedent for the organizational influence of scaffolding proteins and microdomains in neuronal ion channel and GPCR-mediated CREB phosphorylation. For example, the privileged ability of L-type calcium channels to mediate downstream CREB phosphorylation, at the exclusion of other calcium influx mechanisms, is due to the specific sub-cellular localization of these L-channels [64]. Our lab has further shown that caveolin proteins (CAVs) are necessary for the functional isolation of distinct GPCR signaling pathways in both hippocampal and striatal neurons [40], [65], [66]. Future experiments will test the hypothesis that functional microdomains and scaffolding proteins, such as CAVs, mediate signaling specificity in this paradigm.

### CRF Signaling in Striatal Neurons

The interaction between stress and drug addiction is well established. Stress both pre-disposes individuals to drug-addiction, as well as triggers relapse in previously abstinent addicts [1]–[3]. Animal models of stress and drug addiction mirror these results from clinical populations. Stress enhances locomotor responses to drugs of abuse, and enhances acquisition of self-administration [53]. Stress has also been shown to re-activate extinguished CPP for cocaine [4].

CRF is the molecular mediator of many of these effects of stress on drug taking behavior [67]. Acute administration of drugs of abuse activates both hypothalamic and extra-hypothalamic CRF, while CRF administration mimics the effects of stress on drug taking behaviors in animal models. Since the NAc is thought to be the site of many of the neuro-plastic changes that underlie drug addiction, and since CRF has been shown to influence NAc-dependent behaviors and neurons [11], [17], [18], [68], we hypothesize that CRF action in NAc neurons underlies some of the effects of stress on addictive behaviors. It must be noted that NAc is not the sole site of CRF influence on addiction. In the context of addictive processes, CRF has been shown to influence several regions of the mesolimbic dopamine system including the VTA and amygdala [67].

Here we show that CRF can, in fact, directly influence NAc neurons by activating CREB, a transcription factor that is important for the responses to stress [69], as well as drug reward [12]. Our data are consistent with a report showing that stress-facilitation of drug reward depends on CRFR1 and CREB [11]. It has been known for quite some time that many of the plastic changes in NAc neurons required for drug addiction are dependent on transcription of genes and synthesis of new proteins, and that the transcription factor CREB plays a key role [13].

This work, which identifies a novel mechanism of CRF signal initiation at the level of the Gβγ subunit in striatal neurons, adds to a growing literature describing the diverse intracellular signaling pathways that can be activated by CRF. However, it remains to be determined whether CRF-induced Gβγ signaling is unique to the striatum, or whether it represents an essential mechanism of CRF signaling across diverse brain regions. In terms of CRF action in the striatum, our working hypothesis is that stress-induced release of CRF into NAc could lead to CREB activation via the pathway identified here, thus “priming” these neurons for CRE-dependent transcription following a robust drug-induced stimulus. Consequently, CRF may shift the transcriptional balance towards the expression of genes that are required for the plastic changes that characterize addiction, thereby facilitating addictive behaviors. Future work will be aimed at determining the extent by which CRF activation of this Gβγ pathway underlies the physiological and behavioral effects of CRF *in vivo*.

### Conclusion

CRF was first described as the neuropeptide that mediated the HPA response to stress. Since then, CRF has been shown to modulate a wide variety of brain regions outside of the traditional HPA axis, including the NAc. Here we report a novel molecular signaling pathway in striatal neurons in which CRF leads to a Gβγ- and MAPK-dependent increase in CREB phosphorylation. This pathway not only sheds light on CRF signaling by characterizing a novel, neuronal CRF initiated signaling pathway, but also reveals novel potential targets for altering the effects of CRF on a variety of behaviors.

## Materials and Methods

### Neuronal Cell Culture

Striatal neurons were cultured from 1- to 2-d-old rat pups as previously described [65], [70], using a protocol approved by the Animal Care and Use Committee at the University of Minnesota. Chemicals and drugs were obtained from Sigma (St. Louis, MO) or Tocris (Ellisville, MO) unless otherwise noted. Following decapitation, the striatum was isolated in cold HBSS containing 20% fetal bovine serum (FBS; HyClone; Logan, UT), 4.2 mM NaHCO_3_, and 1 mM HEPES, pH 7.35 at 300 mOsm. Tissue was washed before a 5 min digestion in trypsin solution (type XI; 10 mg/mL) containing 137 mM NaCl, 5 mM KCl, 7 mM Na_2_HPO_4_, 25 mM HEPES, and 1500 U of DNase at pH 7.2 and 300 mOsm. For dorsal striatum and central striatum enriched cultures, a coronal section was made at the level of the striatum. The dorsal and ventral portions were identified using the anterior commissure, and then transected using a tungsten needle. Following additional washes, the tissue was dissociated, and the cell suspension was pelleted twice before being plated (6×10^4^ cells per well) on Matrigel (BD Biosciences; San Jose, CA)-treated 10 mm coverslips. Cells were incubated at RT for 15 min. One mL of MEM (Invitrogen; Carlsbad, CA) containing 28 mM glucose, 2.4 mM NaHCO_3_, 0.0013 mM transferring (Calbiochem; La Jolla, CA), 2 mM glutamine, 0.0042 mM insulin, 1% B-27 Supplement (Invitrogen), and 10% FBS was added to each well. Forty-eight hrs later, cells received 1 mL of identical media containing 4 µM cytosine 1-B-D-arabinofuranoside (to inhibit glial mitosis) and 5% FBS. To limit our analysis to GABAergic medium spiny neurons, we excluded large cholinergic interneurons from our analysis based on size.

### Drugs

Also see Table S1. Drugs were obtained from Tocris unless noted. Tetrodotoxin (TTX; 1 µM), D(-)-2-amino-5-phosphonopentanoic acid (AP-5; 25 µM), corticotropin releasing factor (CRF; 40 nM), astressin (100 nM), CP154526 (100 nM), K41498 (10 nM), antisauvagine-30 (100 nM); stressin-1 (STR; 70 nM), H89 (2 µM), forskolin (12.5 µM), PKI, 14–22 amide myristoylated (1 µM), KT5720 (1 µM), cAMPS-Rp, triethylammonium salt (10 µM), SQ22536 (90 µM), isoproterenol (10 µM), IBMX (75 µM), KN-93 (2 µM), nifedipine (5 µM), U0126 (10 µM), PD98059 (25 µM), K252a (100 nM), 10-DEBC (5 µM), H-1152 (500 nM), GW-5074 (1 µM), Gallein (75 µM), pertussis toxin (PTX; 500 ng/mL), cholera toxin (CTX; 500 ng/mL; Sigma), M119 (5 µM; gift from Dr. Kirill Martemyanov), BAPTA-AM (5 µM; Molecular Probes; Eugene, OR), methyl-β-cyclodextrin (MβCD; 10 mM; Sigma).

### Immunocytochemistry

Protocols followed are those described previously, using a well-characterized commercially available monoclonal antibody directed against the Ser133 phosphorylated version of CREB (see below) [65], [66], [70]–[72]. Briefly, cultured striatal neurons (8–10 d.i.v.) were incubated in a Tyrode's solution containing TTX (1 µM) and AP-5 (25 µM) at room temperature for 1.5 hr. Unless noted otherwise, cell stimulations were performed as follows: vehicle (15 min); agonist stimulations were 15 min, antagonist exposure was 30 min prior to agonist stimulation, except PTX and CTX (18 hr pre-treatment), and concurrently with agonist stimulation. Cells were then fixed for 10 min using ice-cold 4% paraformaldehyde (Electron Microscopy Sciences; Ft. Washington, PA) in PBS containing 4 mM EGTA. Following wash, cells were permeabalized in 0.1% Triton X-100 (VWR Scientific; West Chester, PA) for 5 min. Following an additional wash, cells were incubated at 37°C in block solution (1% IgG-Free BSA and 2% Goat Serum (Jackson ImmunoResearch; West Grove, PA) in PBS) for 30 min. Primary antibody incubation consisted of a 1 hr incubation at 37°C in block solution containing a monoclonal antibody directed against the Ser-133 phosphorylated form of CREB (pCREB 10E9, 1∶1000; Upstate Biotechnology, Lake Placid, NY) and to identify individual cell morphology, a polyclonal antibody targeting microtubule-associated protein 2 (MAP2, 1∶1000; Upstate). For CREB staining (Figure S1), a rabbit monoclonal antibody directed against total CREB (CREB 48H2, 1∶1000; Cell Signaling, Boston, MA) and a mouse monoclonal antibody directed against α-tubulin (236–10501; Invitrogen) were used. Cells were then washed before being incubated in block solution containing FITC- and CY5-conjugated secondary antibodies (1∶200; Jackson ImmunoResearch). Following a final wash, cells were mounted using FluorSave (Calbiochem). Nuclear fluorescent intensities for pCREB or CREB (approximately 25 cells per group) were acquired using a Leica DM5500Q confocal system. Data were quantified with Leica LAS AF (version 1.9.0; Leica).

The confocal excitation and detection settings for each experiment were determined using coverslips stimulated for 5 min with 60 mM potassium (60 K) to establish a “ceiling” for pCREB intensity. A baseline was determined via the vehicle (NS) group, so that each assay has an internal control. Inter-coverslip variability was accounted for by subjecting 2–3 coverslips to each treatment. Data were acquired in random order by a blind observer. Neurons were readily discriminated from glia via size and morphology (see Figure 1 and Figure S1) and selected randomly using α-tubulin or MAP2 fluorescence, allowing the experimenter to remain blind to the CREB or pCREB signal. Images were captured through the approximate midline of each neuron. To analyze pCREB fluorescence intensity, the α-tubulin or MAP2 staining was used to draw a region of interested (ROI) of interest outlining the nucleus of each neuron. The ROI was then transferred to the CREB or pCREB image, and average fluorescence intensities within the nucleus were noted. All images were background subtracted from an area devoid of neuronal α-tubulin or MAP2 staining, with each experiment being performed at least three times to verify results.

### cAMP Assay

We measured cAMP concentrations in cultured striatal neurons (8–10 d.i.v.) using a Parameter cAMP kit (R&D Systems; Minneapolis, MN) with a mean minimum detectable dose of 1.50 pmol/mL (manufacturer protocol). In all cAMP assays, striatal neurons were incubated in a Tyrode's solution containing TTX (1 µM) and APV (25 µM) for 1.5 hours, and then switched into an identical solution containing IBMX (75 µM). When indicated, methyl-β-cyclodextrin (MβCD; 10 mM) was included with IBMX. For MβCD experiments, 4 µM CRF was used for 15 min (Figure 6C). Stimulations were performed in the presence of the above compounds for the time-points indicated in “Results” with isoproterenol (10 µM) or CRF. Immediately following stimulation, neurons were washed with ice-cold PBS and then lysed with 215 µL ice-cold lysis buffer. Samples were stored overnight at -20°C before being processed according to manufacturer instructions. A Bio-Rad microplate reader (model 680) was used to measure concentrations of cAMP. Lysate from individual coverslips were placed in separate wells (n∼4 wells/group). Each experiment was performed in triplicate to verify results.

### Neuronal Transfection

Cultured striatal neurons were transfected at 7–8 d.i.v. with 1 ug/DNA/coversip using a calcium-phosphate method that results in >95% of the transfected cells being neurons [70].

### Statistics

Experiments were analyzed using ANOVAs and Bonferroni's Multiple Comparison *post hoc* test, or nonlinear curve fits using Prism 4.03 (GraphPad Software, La Jolla, CA). Statistically different groups are denoted by different alphabetical characters in corresponding bar graphs. P-values <0.05 were considered *a priori* as significant. Data are presented as mean ± SEM.

## Supporting Information

Figure S1 **CRF does not change total CREB.** (A) Immunolabeled confocal images of cultured striatal neurons from 1- to 2-day old rat pups with α-tubulin (green) and total CREB (red). Neurons stimulated with CRF (40 nM) for 15 min showed no change in total CREB staining relative to vehicle stimulated control neurons (NS; Scale Bar  =  20 µm). (B) Quantification of immunostaining revealed no effect of CRF on total CREB staining (P = 0.67, Student's two-tailed T-test).
(TIF)

Table S1 **List of inhibitors used in this study** [**31]**, [32], [36], [37], [71], [73]–[88****] **.**
(XLSX)
